# The Odyssey of Hsp60 from Tumor Cells to Other Destinations Includes Plasma Membrane-Associated Stages and Golgi and Exosomal Protein-Trafficking Modalities

**DOI:** 10.1371/journal.pone.0042008

**Published:** 2012-07-25

**Authors:** Claudia Campanella, Fabio Bucchieri, Anna M. Merendino, Alberto Fucarino, Giosalba Burgio, Davide F. V. Corona, Giovanna Barbieri, Sabrina David, Felicia Farina, Giovanni Zummo, Everly Conway de Macario, Alberto J. L. Macario, Francesco Cappello

**Affiliations:** 1 Dipartimento BIONEC, Sezione di Anatomia Umana, University of Palermo, Palermo, Italy; 2 Istituto Euro-Mediterraneo di Scienza e Tecnologia, Palermo, Italy; 3 Dipartimento STEMBIO, University of Palermo, Palermo, Italy; 4 Dulbecco Telethon Institute, Palermo, Italy; 5 Istituto di Biomedicina e Immunologia Molecolare, C.N.R., Palermo, Italy; 6 Department of Microbiology and Immunology, School of Medicine, University of Maryland at Baltimore, and IMET, Baltimore, Maryland, United States of America; University of Campinas, Brazil

## Abstract

**Background:**

In a previous work we showed for the first time that human tumor cells secrete Hsp60 via exosomes, which are considered immunologically active microvesicles involved in tumor progression. This finding raised questions concerning the route followed by Hsp60 to reach the exosomes, its location in them, and whether Hsp60 can be secreted also via other mechanisms, e.g., by the Golgi. We addressed these issues in the work presented here.

**Principal Findings:**

We found that Hsp60 localizes in the tumor cell plasma membrane, is associated with lipid rafts, and ends up in the exosomal membrane. We also found evidence that Hsp60 localizes in the Golgi apparatus and its secretion is prevented by an inhibitor of this organelle.

**Conclusions/Significance:**

We propose a multistage process for the translocation of Hsp60 from the inside to the outside of the cell that includes a combination of protein traffic pathways and, ultimately, presence of the chaperonin in the circulating blood. The new information presented should help in designing future strategies for research and for developing diagnostic-monitoring means useful in clinical oncology.

## Introduction

Molecular chaperones, many of which are Heat shock proteins (Hsps), are important players in protein homeostasis and cell and tissue physiology, as well as in protection against stressors [1]. Hsps intervene not only in protein folding, refolding, trafficking and degradation but also in the regulation of cell growth and differentiation, apoptosis and cell-to-cell crosstalk, inflammation, and tissue repair [1], [2].

The importance of chaperones has come into focus in the last few years because it has been realized that they can be pathogenetic factors in a variety of conditions named chaperonopathies [1]. Among these pathologies there are various forms of cancer (chaperonopathies “by mistake” or “by collaborationism”), in which chaperones are normal but work in favor of the tumor rather than protect the patient [3]. In these chaperonopathies by mistake, the chaperone involved enhances tumor cell survival and growth by, for example, inhibiting apoptosis and the anti-tumor immune response, or by promoting neoangiogenesis [4]–[6].

That Hsp60 is actively involved in carcinogenesis has been suspected for many years. Its levels have been found increased in a number of neoplasms in which it may be found intra- and peri-cellularly, and in circulation [for a review, see 7]. In a recent work, we showed for the first time, that human tumor cells can secrete Hsp60 via exosomes [8], extracellular vesicles with important roles in immune system activation during cancer progression [9], [10]. Exosomes are released from normal and tumor cells by multivesicular bodies (MVB), a membranous intracellular complex generated by the fusion of membrane-derived early endosomes with other intracellular vesicles [9], [10]. Lipid rafts and Golgi vesicles participate in MVB formation [9], [11]. The presence of Hsp60 in tumor derived exosomes raised questions such as: 1) Does cytosolic Hsp60 reach the plasma membrane in tumor cells? 2) Are lipid rafts involved in Hsp60 membrane trafficking? 3) What is the location of exosomal Hsp60? 4) Is it integrated in the exosomal membrane? and 5) Is the Golgi involved in Hsp60 secretion from tumor cells, as well? The present work deals with these questions. The results allowed us to outline the possible route that Hsp60 follows inside tumor cells before its secretion into the extracellular space, and provide clues for developing new antitumor treatments centred on the chaperonin.

## Results

### Hsp60 is Present in the Tumor-cell Plasma Membrane and in Lipid Rafts

**Figure 1:**
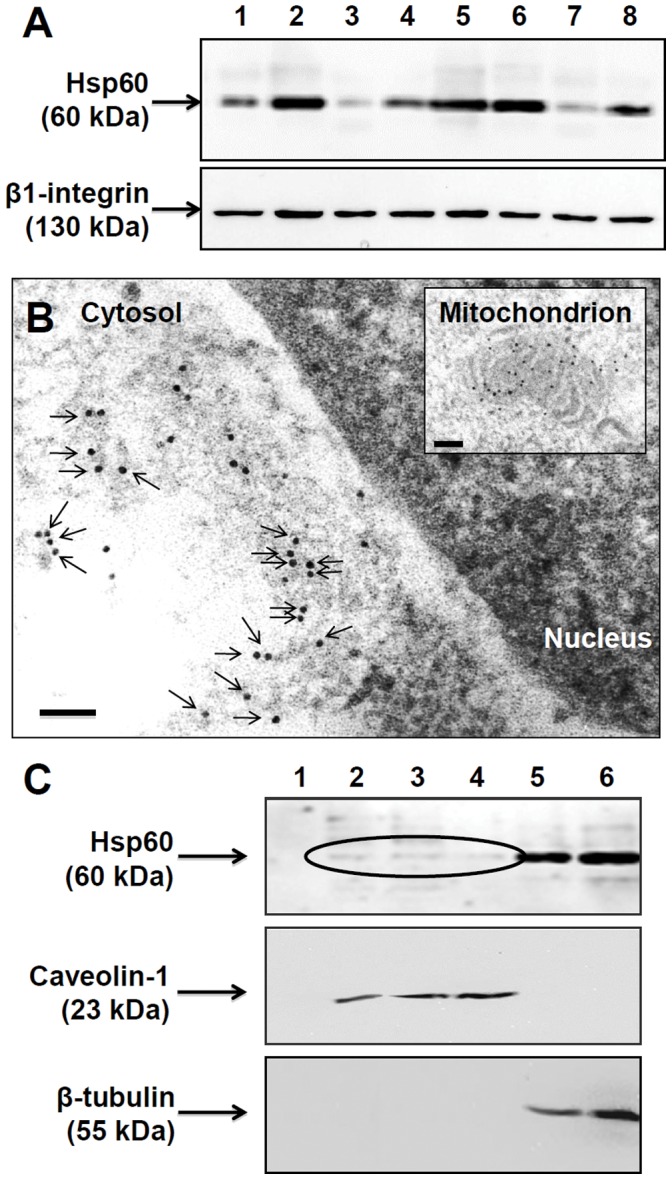
Hsp60 is present in the plasma membrane and lipid rafts of tumor cells. A. Western blotting for Hsp60, and β1-integrin as loading control, of isolated plasma membrane. Each lane represents a separate experiment. Lanes 1–4, membrane from H292 tumor cells; 5–8, membrane from A549 tumor cells. **B.** Transmission electron microscopy-Immunogold demonstration of Hsp60 (black dots) in H292 cells. Arrows indicate the Hsp60 molecules that are close or onto the cell membrane. The inset shows the typical pattern of Hsp60 in a mitochondrion, which serves as a positive internal control. Bar: 100 nm. **C:** Western blotting for Hsp60, caveolin-1, and β-tubulin in isolated lipid rafts from H292 tumor cells. Hsp60 is present in lanes 2 to 6; caveolin-1 (marker for lipid rafts) is present in lanes 2 to 4; and β-tubulin (cytosolic protein) is present in lanes 5 and 6.

Hsp60 was found in the plasma membrane fractions isolated from H292 (human lung mucoepidermoid) and A549 (human lung adenocarcinoma) cells (Fig. 1A). In contrast, Hsp60 was not present in the plasma membrane fractions from the 16HBE line, which derives from normal human bronchial epithelial cells (data not shown). The levels of Hsp60 in the plasma membrane fractions obtained from the tumor cells H292 and A549 were not the same in all experiments, which could reflect different stages of the chaperonin trafficking. The presence of Hsp60 in the plasma membrane of the tumor cells was also demonstrated by Transmission Electron Microscopy (TEM)-immunogold (Fig. 1B). Hsp60 was localized not only in the plasma membrane but also in the cytosol, very close to the plasma membrane, suggesting active movement of the chaperonin in this area, possibly toward, and also away from, the plasma membrane.

Since Hsp60 was present in the plasma membrane fraction obtained from tumor cells, we performed experiments to determine if the chaperonin occurred in the lipid rafts fraction obtained from the same tumor cells. The results showed that Hsp60 was present in the lipid rafts fractions obtained from H292 (Fig. 1C) and A549 (not shown) cells. The occurrence of Hsp60 in the lipid rafts fractions of tumor cells is in agreement with our previous data [8], showing that methyl-b-cyclodextrin (MBC), a lipid-raft pathway inhibitor, reduced the quantity of Hsp60 released extracellularly from tumor cells. Altogether, the data suggest that lipid rafts receive and then internalize Hsp60 from the plasma membrane to intracellular vesicles, i.e., the microvesicular bodies (MVB), thus participating in Hsp60 trafficking at the plasma membrane level.

### Hsp60 is Integrated in the Membrane of Exosomes Derived from Tumor Cells

We have already demonstrated that Hsp60 occurs in exosomes produced and released by tumor cells [8]. In this work, we investigated if Hsp60 localizes in the exosomal membrane. Clarification of this point is important because it is known that proteins integrated in the exosomal membrane are putative ligands for receptors present on the target cells, while proteins contained inside exosomes are discharged into the cytosol of target cells [9], [10], [12], [13].

**Figure 2:**
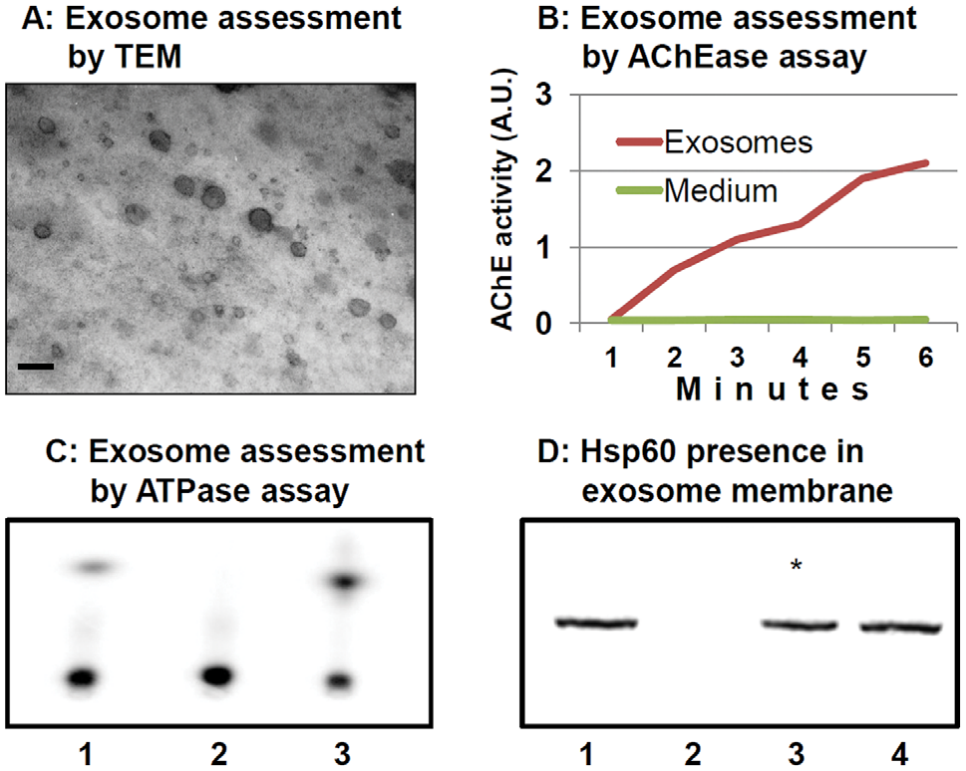
Hsp60 is integrated in the membrane of exosomes from tumor cells. A–C. Illustrative data on the exosome preparations utilized in this work. **A**, Transmission electron microscopy demonstrates that the dimension of isolated vesicles is equal to, or smaller than 100 nm, which is consistent with exosomes. Bar: 100 nm. **B** and **C**, acetylcholinesterase (AChEase) and ATPse enzymatic activities, respectively, typical of isolated exosomal vesicles, compared to control (conditioned culture medium). In B, the solid line represents data from exosomes, and the broken line represents results from conditioned culture medium. Vertical axis, AChEase activity in arbitrary units (AU), reflecting 412 nm absorbance; horizontal axis, time of reaction in minutes. In C, 1, marker (positive control); 2, conditioned culture medium; 3, exosomes. **D.** Treatment with sodium carbonate alone (lane 3, asterisk), or in association with proteinase K buffer (lane 4), does not remove Hsp60 from the exosomes, whereas treatment with Proteinase K does (lane 2). Lane 1, untreated exosomes (positive control).

We performed experiments with exosomes purified from both H292 (Fig. 2) and A549 (not shown) cells. We characterized the purified exosomes by transmission electron microscopy (TEM) (Fig. 2A) and by determining AChEase and ATPase enzymatic activities (Fig. 2B and C), according to established criteria [9]–[12]. Subsequently, we looked for Hsp60 in the exosomal membrane. As shown in Fig. 2D, Hsp60 was integrated in the exosomal membrane. Our results do not exclude the presence of Hsp60 also in the exosomal lumen.

### Hsp60 Occurs also in the Golgi Apparatus

**Figure 3:**
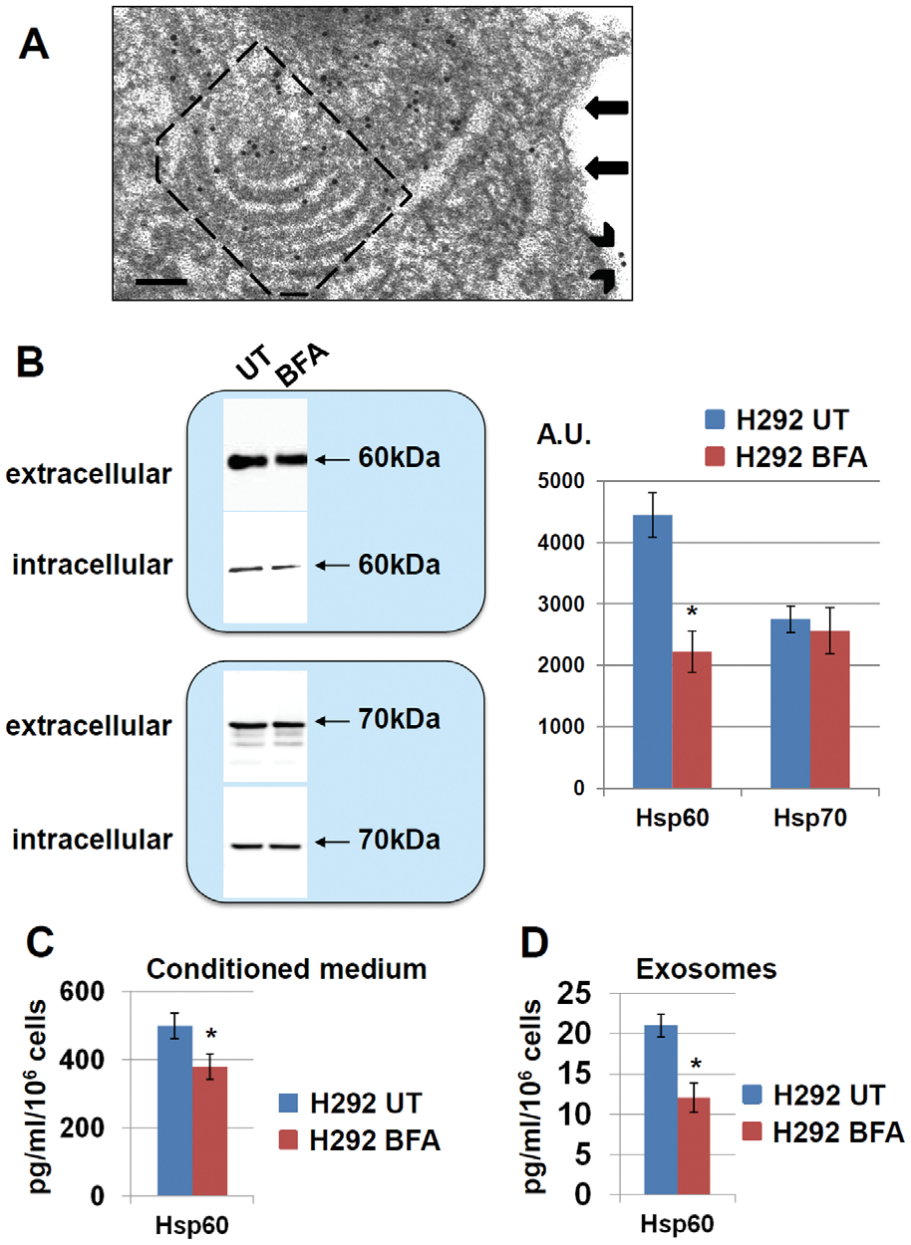
Golgi involvement in Hsp60 secretion from tumor cells. A. TEM**-**Immunogold shows Hsp60 (black dots) in tumor cells, including in the Golgi (framed by a dashed rectangle). The arrows show the plasma membrane and arrowheads indicate Hsp60 in it. Bar: 100 nm. **B.** Extracellular levels of Hsp60 decrease after Brefeldin A (BFA) treatment of the tumor cells, as measured in immunoprecipitates from the conditioned medium (left upper panel). In contrast, Hsp70 levels are not influenced by BFA treatment (left lower panel). Right hand panel: histograms showing densitometric measurements of the Western blots to the left. UT, untreated; A.U.: arbitrary units; asterisk indicates p<0.005. **C.** ELISA results demonstrate a reduction of Hsp60 levels in the conditioned culture medium from tumor cells after BFA treatment. UT: untreated. **D.** ELISA results show a reduction of Hsp60 levels in exosomes after BFA treatment of the tumor cells. UT: untreated. Asterisks in C and D, p<0.05. Error bars represent SD.

Since soluble free Hsp60 has been found in the circulating blood of patients with some types of cancer [14], we investigated if this chaperonin is secreted from H292 (Fig. 3) and A549 (not shown) cells also via Golgi. We found Hsp60 inside the Golgi of tumor cells by TEM-Immunogold (Fig. 3A). In addition, we performed experiments using brefeldin A (BFA), a recognized selective Golgi inhibitor, and we found that Hsp60 levels in the extracellular medium of cultured tumor cells (conditioned medium) was significantly reduced by treating the cells with BFA, as shown by Western blotting (Fig. 3B) and ELISA (Fig. 3C). In contrast, BFA did not reduce the concentration of extracellular Hsp70 (Fig. 3B), another well studied chaperone whose extracellular effects have been documented [2].

Interestingly, we found a reduction of exosomal Hsp60 levels after BFA treatment (Fig. 3D). While this finding was not examined further, it may be taken to indicate that Golgi-derived vesicles may usher Hsp60 toward MVB and then exosomes.

## Discussion

Hsp60 levels are increased in a variety of tumors and pre-neoplastic lesions *in vivo*, providing a potential means of diagnosis and disease monitoring [reviewed in 7 and 15]. A role for Hsp60 in carcinogenesis has been postulated by various authors but it is not yet fully understood [7], [16]–[18]. Consequently, there is interest in elucidating, for instance, the effects of Hsp60 (and also other Hsps) on some types of cells such as those of the immune system during carcinogenesis *in vivo*, particularly when the chaperones gain the extracellular space and the circulation [15], [19]–[21]. Since the story must begin when the Hsp60 molecule exits the tumor cell, we performed experiments to determine what secretion mechanisms are involved at this early stage.

In previous work, we showed that Hsp60 is present in the exosomes released from human tumor cells and that this release is significantly reduced after treating the tumor cells with inhibitors of the exosome and lipid-raft protein-trafficking pathways [8]. The data opened new research avenues pertaining to the location of Hsp60 in the tumor cell plasma membrane and lipid rafts, and in the exosomes themselves. Furthermore, it was pertinent to ask the question whether also the Golgi apparatus plays any role in the Hsp60 movements inside the cell and toward the extracellular space. All these are key aspects of Hsp60 physiology that require elucidation in order to establish the chaperonin’s potential as biomarker for diagnosis, disease monitoring and treatment, and to design novel anti-cancer therapeutic strategies centred on the chaperonin. Along these lines, in the present work we investigated the presence of Hsp60 in the plasma membrane of tumor cells, analogously to what has been done for other Hsps [22]. Membrane Hsps have been considered potentially convenient targets for specific antibodies in therapeutic anti-tumor immunity [23]. Our data provide evidence that Hsp60 could also be useful in manipulating anti-tumor immunity with therapeutic purposes. In this regard, it is particularly important to bear in mind that Hsp60 in normal cells is present mostly intracellularly with little presence on the plasma membrane [24]–[27], which is in contrast with tumor cells, at least in some types of tumors [28]–[30]. Therefore, in the latter type of tumors, anti-Hsp60 antibodies would be expected to hit tumor cells preferentially but much less their normal counterparts.

We found that Hsp60 is integrated in the exosome membrane. This novel piece of information is important because it might indicate that Hsp60 in the exosome membrane, like other proteins in a similar location, would serve as a ligand for receptors on the surface of cells targeted by the exosomes, such as cells of the immune system, or tumor cells other than that from which any given exosome originated. It is accepted that immune cells are preferential targets for exosomes as these participate in immune response regulation. Other exosome-bound Hsps, e.g., Hsp70, have been found to recognize receptors on immune system cells [21]. It has recently been reported that HepG2 cells (a human hepatocellular carcinoma cell line) secrete Hsps, including Hsp60, in exosomes [31]. The amount of secreted exosomal Hsp60 increased significantly after treating the HepG2 cells with irinotecan hydrochloride and carboplatin, to which the cells were resistant, in comparison with HepG2 cells stimulated with heat shock or other drugs to which the HepG2 cells were not resistant. The Hsp60-carrying exosomes derived from HepG2 cells treated with the compounds to which they were resistant elicited a stronger cellular immune anti-tumor response compared to exosomes from HepG2 cells treated with other drugs, and this stronger response was accompanied by granzyme B release and plasma-membrane receptor density modification in NK cells. All together these data support the view that Hsp-carrying exosomes from at least some types of tumors and under certain conditions are stimulatory vehicles than can elicit NK-cell bioactivity.

One may speculate that a way to modulate an anti-tumor immune response would be to manipulate exosomes in what regards their abundance in the circulation and Hsp60 cargo and location by controlling, for example, lipid raft-mediated Hsp60 trafficking. The implications for clinical oncology are no doubt potentially far reaching.

In this work, we also collected evidence in favour of a participation of the Golgi in Hsp60 release from tumor cells. Our data suggest that Hsp60, but not Hsp70, secretion from tumor cells involves the Golgi apparatus, in addition to the exosomal pathway as shown in a previous publication [8]. Our data are in agreement with those of another group that recently showed that Hsp60 was present in the Golgi apparatus of malignant cells but not in the Golgi of normal cells and that treatment with Brefeldin A influenced Hsp60 localization and secretion by tumor cells [32]. In addition, our data suggest that the Golgi apparatus could be responsible of Hsp60 import into exosomal vesicles, as well as of Hsp60 secretion of the free, soluble form detected in some tumor cases [14], [33]. Unfortunately, little attention has been given to the potential of Hsp60 as a biomarker to be measured for assessing the clinical status of cancer patients [14]. We hope our data will stimulate further research in this direction.

**Figure 4:**
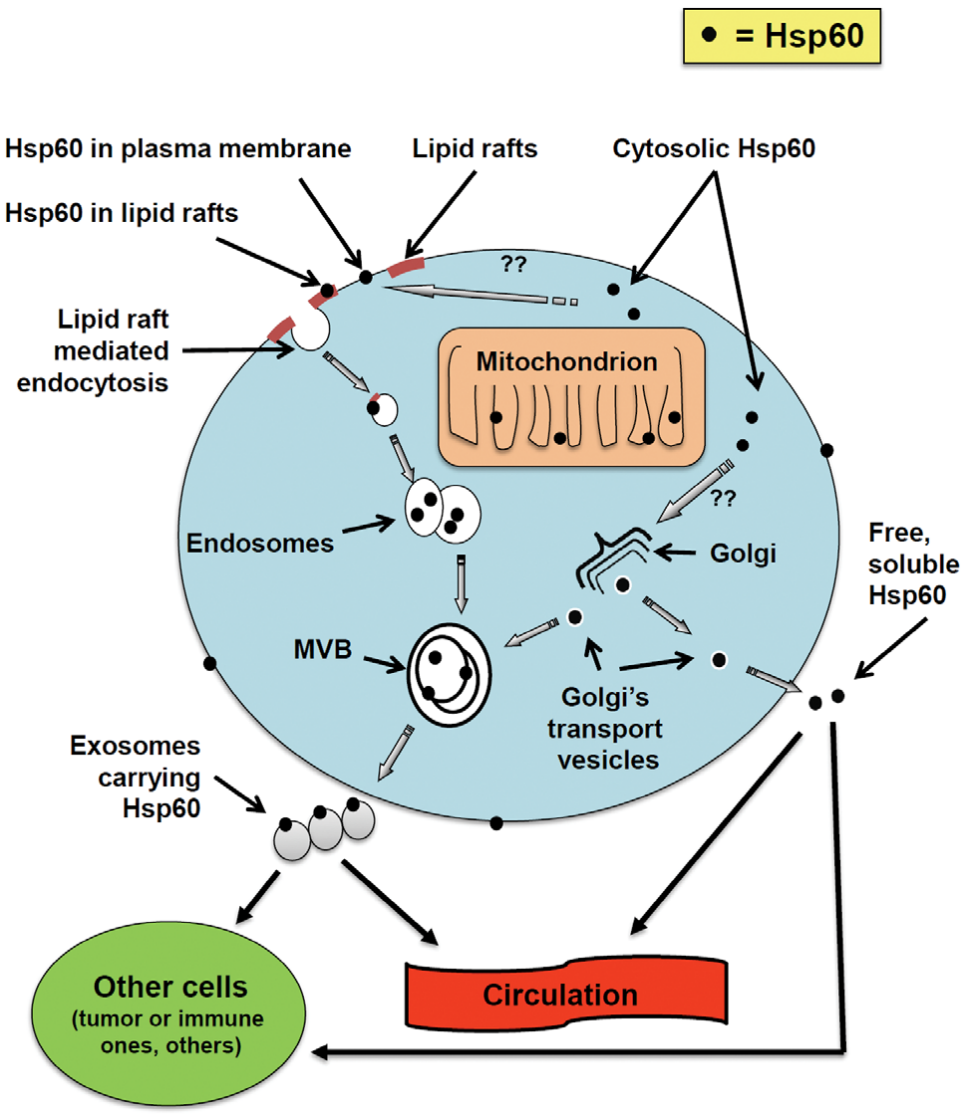
Proposed Hsp60 secretion pathways in a tumor cell. Hsp60 (black dots) that in normal cells localizes mainly in mitochondria, in tumor cells accumulates also in the cytoplasm and, for unknown reasons (post-translational modifications?), reaches the cell membrane and the Golgi. At the membrane, lipid rafts internalize (endocytose) Hsp60 toward multivesicular bodies (MVB) from where it is secreted via exosomes. In these, it is located in the membrane and probably also inside. Hsp60-loaded exosomes thus would reach other cells near and far through the circulation. The Golgi may also participate in Hsp60 secretion via transport vesicles moving to both MVB and the extracellular space. Hsp60 released in the extracellular space by Golgi vesicles (free Hsp60) can thus reach other cells in the vicinity and distant via circulation.

In conclusion, this study sheds new light on the Odyssey of Hsp60 inside the cell in which it originates, its exit from it, and its voyage to its final destination in other cells. As a framework for future research we propose a multistep process, outlined in Fig. 4. This process would involve the plasma membrane, lipid rafts, Golgi, MVB, and exosomes. We still need, for example, to establish why and how cytosolic Hsp60 reaches the plasma membrane, and whether post-translational modifications of the chaperonin are involved. Another possibility that cannot be ruled out at the present time is that Hsp60 does not travel alone but bound together with another molecule (or molecules) and, thus, translocates in ways and to places that would be impossible otherwise. This thought is pertinent, for instance, in considering the mechanism by which Hsp60 goes into the Golgi when it is known that this chaperonin does not have a canonical Golgi-localization signal. Finally, other points to be investigated are whether extracellular Hsp60 is internalized by cells in the vicinity of the cell that secretes it and, if so, whether this internalization involves other molecules.

## Methods

### Cell Cultures

The human lung mucoepidermoid carcinoma H292, the human lung adenocarcinoma A549 and the non-tumor human bronchial epithelial 16HBE cell lines were obtained from the American Type Culture Collection. H292 and A549 were maintained in RPMI 1640 medium; 16HBE was maintained in D-MEM medium. Both media were supplemented with 10% heat-inactivated fetal calf serum and with 2 mM glutamine, 50 U/ml penicillin, and 50 mg/streptomycin. Cells were grown as monolayers and cultured at 37°C, in an atmosphere with 5% CO_2_, in a humidified incubator. Passage numbers of cells used in this study ranged from 12 to 35. All cell culture reagents were purchased from GIBCO BRL LIFE Technologies (Invitrogen, Milan, Italy). All experiments were conducted at least in triplicate.

### Transmission Electron Microscopy (TEM) Immunogold for Hsp60

To detect human Hsp60, we used the following antibodies: polyclonal rabbit anti-Hsp60 (clone H-300, Santa Cruz Biotechnologies, Santa Cruz, CA, USA) diluted 1∶15; and the goat-anti rabbit IgG (H&L) 10-nm gold-conjugated Aurion (Ge Healthcare, Milan, Italy), diluted 1∶30.

After plating for 24 hours, H292 and A549 cells were fixed with 4% paraformaldehyde and 0.5% glutaraldehyde in 100 mM sodium cacodylate buffer (pH 7.4) for 30 min and included the same day. After fixation, samples were rinsed twice with 100 mM sodium cacodylate buffer (pH 7.4), and then dehydrated with ethanol (30%, 50%, 70%). After dehydration cells were embedded into London Resin-white resin (L. R. White, London Resin Co, London, U.K.) inside gelatin capsules at 60°C. The samples were then cut into ultrathin sections. After pre-treatments with BSA for 10 min at 24°C, they were incubated with primary anti-Hsp60 antibody for 1 hour. Following washing in PBS (5 times for 5 min each) the sections were incubated with secondary anti-rabbit antibody for 1 hour. Sections were then washed in PBS and then with distilled water, and were stained with uranyl acetate and subsequently examined by JEOL JEM 1220 TEM at 120 kV.

### Isolation of Plasma Membranes and Lipid Rafts

We used, with some modifications, the technique described by Barbieri et al. [34] for plasma membranes isolation from H292, A549, and 16HBE cells and the method published by Xavier et al. [35] for lipid rafts isolation from H292 and A549 cells.

To isolate plasma membrane, the cells were first swollen in hypotonic buffer (5 mM KC1, 1 mM Mg C1_2_, 20 mM Hepes pH 7.9, and 1 mM Na_3_VO_4_) containing an anti-proteases cocktail for 30 min on ice, then lysed with a tight-fitting pestle of a potter homogenizer (60 strokes). Nuclei were separated by centrifugation at 1000×g for 10 min at 4°C. The supernatant was centrifuged at 100,000×g for 30 min at 4°C to pellet the plasma membrane fractions and yield the cytosolic fractions. The plasma membrane fractions were extracted in buffer A (25 mM Tris, pH 7.5, 250 mM sucrose, 2.5 mM MgC1_2_, 5 mM EGTA, 5 mM EDTA, 1% Triton X-100, 0.5% deoxycholate, 1 mM Na_3_VO_4_ and protease inhibitors) for 30 min on ice, centrifuged at 10,000×g for 30 min at 4°C to pellet debris.

To isolate the lipid raft fractions, cells were lysed in MBS buffer (25 mM MES, 2 mM EDTA pH 8, and 150 mM NaCl) containing 1% Triton X 100 and anti-proteases cocktail for 30 min on ice. The lysates, mixed with an equal volume of 85% sucrose (w/v) in MBS buffer, were placed at the bottom of a polycarbonate ultracentrifuge tube (Beckman Instruments, Palo Alto, CA, USA), overlaid with 2 ml of 35% sucrose and 1 ml of 5% sucrose in MBS buffer containing 2 mM EDTA pH 8 and anti-proteases cocktail and were centrifuged at 100,000×g for 20 hours at 4°C in a SW55 Ti rotor (Beckman Instruments). Six fractions of 550 µl/each were collected from the top of the discontinuous sucrose gradient. The isolated plasma membrane fractions and lipid rafts were used directly in SDS-PAGE and Western blotting, as described below.

### Protein Quantification

Proteins from plasma membrane and lipid rafts preparations were quantified with the Quant-iT TM protein assay kit, using Qubit fluorometer (Invitrogen Molecular Probes, Invitrogen, Milan, Italy), according to the manufacturer’s instructions. The kit is accurate for protein concentrations raging from 12.5 µg/ml to 5 mg/ml.

### Western Blotting

We used the following primary antibodies: anti-Hsp60 monoclonal antibody (clone LK1, Sigma-Aldrich Inc, Milan, Italy), diluted 1∶1,000; anti-Hsp70 monoclonal antibody (clone W27, Santa Cruz Biotechnologies), diluted 1∶1,000; anti-β1-integrin monoclonal (clone N29, Chemicon- Millipore Corporation, Billerica, MA, USA) diluted 1∶1,000; anti-caveolin-1 monoclonal antibody (clone 4H312, Santa Cruz Biotechnologies), diluted 1∶1,000; anti-β-tubulin monoclonal antibody (clone TUB 2.1, Sigma-Aldrich Inc), diluted 1∶1,000. Western blotting was performed as previously described [8]: briefly, 40 µl of samples was added to 4x Laemmli buffer and heated for 5 min at 95°C. Proteins were resolved by 12% SDS PAGE along with molecular weight marker (Bio-RAD Laboratories, Milan, Italy). Proteins were then transferred to nitrocellulose membranes. The membranes were blocked with 5% fat milk, and probed for 12 hours with the primary antibodies, followed by incubation with horseradish peroxidase-conjugated second antibody (Amersham Biosciences, Milan, Italy) diluted 1∶5,000. Blots were detected using Supersignal West Femto, according to the manufacturer’s instructions (Pierce, Milan, Italy) and chemiluminescent signals were recorded with ChemiDoc XRS imager (Bio-RAD Laboratories). Densitometric analysis of plots was performed using the NIH Image J analysis program (National Institutes of Health, Bethesda, MD, USA). Western blotting for Hsp60 and anti-β1-integrin detection in plasma membrane was performed loading 100 µg of proteins in each lane. Western blotting for Hsp60, caveolin-1, and β-tubulin detection in lipid rafts was performed loading 50 µg of proteins in each lane.

### Isolation of Exosomal Fractions

1. Conditioned medium: Eighty ml of conditioned medium from 70–80×10^6^ cells (H292 or A549) were collected after 24 hours of culture in serum-free medium, and centrifuged (800×g for 10 min) at 4°C to eliminate cells and debris.
2. Purification of exosomes: Exosomes were isolated as previously described [8]: briefly, 50 ml of cell- and debris-free medium was collected on ice and centrifuged at 13,000×g for 20 min at 4°C to bring down and eliminate small cellular debris and mitochondrial contaminants. The supernatant was collected and exosomes were separated from it by centrifugation at 110,000×g for 2 hours at 4°C in a Beckman 60 Ti rotor; the pellet (called “exosomal fraction”) was collected and washed once in phosphate-buffered saline (PBS), resuspended in 100 µl of PBS containing proteases inhibitor, and stored at −80°C until use.
3. Assessment of exosomes’s quality by transmission electron microscopy (TEM): Exosome pellets purified from the cell lines studied were first examined by TEM to ascertain the presence of exosomal vesicles, whose diameter has to be 100 nm or less [9], [10]. Pellets were resuspended in residual fluid from PBS wash, followed by addition of 100 µl freshly made fixative (2.5% glutaraldeyde in PBS) to preserve vesicle structure and morphology. Preparations were mounted on formvar nickel 300-mesh grids by layering grids over 10 ml drops of exosome preparations for 10 min at 24°C. Grid-mounted preparations were stained with uranyl acetate and lead citrate, and subsequently examined with JEOL JEM 1220 TEM at 120 kV.
4. Assessment of exosomes’s quality by acetylcholine esterase (AChEase) and ATPase assays: We measured the activity of AChEase and ATPase, two enzymes that are considered reliable markers for these vesicles [9], [10]. AChEase assay was performed as previously described [8]: briefly, 15 µl of the exosomal preparation was suspended in 100 µl of PBS and incubated with acetylcholine (1.25 mM) and 5,5′–dithio-bis-(2-nitrobenzoic) acid (0.1 mM) in a final volume of 1 ml. The incubation was carried out in cuvettes at 37°C and the change in absorbance at 412 nm was monitored every 5 min for 30 min by a plate reader (Titertek Multiscan MCC/340, Flow Laboratories, Lugano, Switzerland). ATPase assay was performed as previously described [36]: briefly, the exosome fraction was added to ATPase buffer (6.6 mM HEPES (pH 7.6), 0.66 mM EDTA, 0.66 mM 2-mercaptoethanol, 0.033% NP-40, 1.1 mM MgCl_2_, 33 µM ATP, 5 µCi (γ-33P) ATP-3000 mmol^−1^ (Ge Healthcare)), and 100 ng of plasmidic DNA was used as substrate. The cell culture medium was used as control and it was setup in parallel. The reactions were incubated for 30 minutes at 24°C. Unreacted ATP and free y-phosphate were separated by thin layer chromatography using TLC cellulose (Merck Millipore, Milan, Italy). ATP hydrolysis quantification was detected with a Bio-RAD, Personal Molecular Imager FX System.

### Search for Hsp60 Presence in the Exosomal Membrane

With the aim to determine whether the Hsp60 was embedded in the exosomal membrane, we used H292 and A549 cells and applied, with some modifications, the techniques described by Fujiki et al. [37] for sodium carbonate treatment of isolated membranes, and the method by Hardy et al. [38] for proteolysis of extracellular membranes by proteinase K treatment. The sodium carbonate treatment flattens the exosomal membrane and releases proteins that are not integrated into the membrane [37], while the proteinase K treatment disrupts the exosomal membrane, releasing all proteins contained in it [38]. Briefly, sodium carbonate treatments were performed treating exosomes with 1 M Na_2_CO_3_, pH 11.5, for 30 min at 37°C. Proteinase K treatments was performed resuspending a pool of carbonate-treated exosomes in 7 mM proteinase K plus a buffer made of 10 mM EDTA and 5 mM N-ethylmaleimide (NEM). Finally, another pool of carbonate-treated exosomes was resuspended only in proteinase K buffer (10 mM EDTA plus 5 mM NEM); this treatment does not modify the results of carbonate-treatment alone and was made as internal control. After treatments, the exosomes were collected by high speed centrifugation for 2 hours at 4°C in a Beckman rotor Ti50. The exosomes fractions obtained were used for Western blotting.

### Treatments with Golgi Inhibitor

We reproduced, with some modifications, the technique described by Hardy et al. [38] for Brefeldin A (BFA) treatments, using H292 and A549 cells. Briefly, cells were seeded in serum-free medium under the same conditions described earlier and treated with 1 µg/ml of BFA for 30 min, followed by a 4-hour recovery period. Extracellular medium was used for immunoprecipitation or for purification of exosomes, as described before.

### Immunoprecipitation

Immunoprecipitation of proteins obtained from extracellular conditioned medium was performed as previously described [8]: briefly, to assess the presence and quantity of Hsp60 in the conditioned medium after BFA treatments, 500 mg of proteins were incubated with 3 mg of anti-Hsp60 monoclonal antibody (clone LK1, Sigma-Aldrich Inc.) at 4°C for 2 hours, followed by incubation with 20 µl protein-A Sepharose at 4°C for 12 hours. Subsequently, the incubation mixture was centrifuged in a microcentrifuge at 14,000×g for 30 seconds at 4°C, the pellet was collected and resuspended in 1×lysis buffer (20 mM Tris-HCl (pH 7.5), 150 mM NaCl, 1 mM Na2EDTA, 1 mM EGTA, 1% Triton, 2.5 mM sodium pyrophosphate, 1 mM b-glycerophosphate, 1 mM Na3VO4, 1 µg/ml leupeptin) and centrifuged again. This procedure was repeated three times. The last pellet was solubilised by boiling into 2×sample buffer (2% SDS, 10% glycerol, 100 mM DTT, 60 mM Tris-HCl (pH 6.8) and 0.001% bromophenol blue), and used for SDS-PAGE as described (see “Western blotting” Section, above). The same protocol, using anti-Hsp70 monoclonal antibody (clone W27, Santa Cruz Biotechnologies), was applied to assess Hsp70 levels.

### ELISA

ELISA tests were performed as previously described [8]: briefly, quantitative comparisons of Hsp60 levels in conditioned media and exosomes from H292 and A549 cells were performed using a commercial Hsp60 EIA Kit from Stressgen Assay Designs Inc. (Ann Arbor, MI, USA). The results obtained with this assay were normalized for cell number and expressed as pg/mL/10^6^ cells.

### Statistical Analysis

Data are presented as the mean ± SD of triplicate determinations. Comparisons between groups were performed using the unpaired samples Student’s t test. A p value <0.05 was considered statistically significant.
